# Identification of Hub Genes in the Remodeling of Non-Infarcted Myocardium Following Acute Myocardial Infarction

**DOI:** 10.3390/jcdd9120409

**Published:** 2022-11-22

**Authors:** Lingxiao Wang, Yan Zhang, Mengjie Yu, Wuzhou Yuan

**Affiliations:** The Center for Heart Development, State Key Laboratory of Development Biology of Freshwater Fish, College of Life Sciences, Hunan Normal University, Changsha 410081, China

**Keywords:** acute myocardial infarction, non-infarcted myocardium, extracellular matrix, macrophage-driven inflammation, bioinformatics, hub genes

## Abstract

(1) Background: There are few diagnostic and therapeutic targets for myocardial remodeling in the salvageable non-infarcted myocardium. (2) Methods: Hub genes were identified through comprehensive bioinformatics analysis (GSE775, GSE19322, and GSE110209 from the gene expression omnibus (GEO) database) and the biological functions of hub genes were examined by gene ontology (GO) functional enrichment and Kyoto Encyclopedia of Genes and Genomes (KEGG) pathway enrichment. Furthermore, the differential expression of hub genes in various cell populations between the acute myocardial infarction (AMI) and sham-operation groups was analyzed by processing scRNA data (E-MTAB-7376 from the ArrayExpress database) and RNA-seq data (GSE183168). (3) Results: Ten strongly interlinked hub genes (*Timp1, Sparc, Spp1, Tgfb1, Decr1, Vim, Serpine1, Serpina3n, Thbs2,* and *Vcan*) were identified by the construction of a protein–protein interaction network from 135 differentially expressed genes identified through comprehensive bioinformatics analysis and their reliability was verified using GSE119857. In addition, the 10 hub genes were found to influence the ventricular remodeling of non-infarcted tissue by modulating the extracellular matrix (ECM)-mediated myocardial fibrosis, macrophage-driven inflammation, and fatty acid metabolism. (4) Conclusions: Ten hub genes were identified, which may provide novel potential targets for the improvement and treatment of AMI and its complications.

## 1. Introduction

As early as the Global Burden of Disease report from 1998 (GBD 1998), acute myocardial infarction (AMI) has been recognized as the most frequent potentially fatal cardiac event worldwide [1]. Despite the development of modern medicine and considerable advances made in AMI treatment, morbidity and mortality rates are still increasing [2,3]. In AMI, acute coronary occlusion results in sustained ischemia and ultimately in irreversible myocardial injury [4,5]. In turn, this tissue damage leads to complications, such as cardiogenic shock, infarct extension, re-infarction, and fatal arrhythmia, which are the leading causes of death following AMI [3]. Accordingly, early detection and treatment are critical to limit infarct damage and associated complications.

Several biomarkers have been identified for AMI diagnosis, including cardiac troponin (cTn) isoforms I and T [6]. However, the diagnostic efficacy of cTn is limited to a relatively brief temporal window, so early ischemic events and complications after AMI may be missed [6]. The primary goals of acute treatment for AMI are reperfusion and restoration of normal heart rhythms and a variety of anticoagulant, fibrinolytic, anti-hypertensive, anti-hyperlipidemic, and anti-arrhythmia drugs, as well as percutaneous intervention methods, are available. However, these options may have adverse effects or limited efficacy for advanced cases and complications after AMI [3,6]. Therefore, the optimization of existing treatment strategies and the development of more effective and accessible diagnostic and therapeutic targets are essential for combatting AMI.

With advances in gene chips, high-throughput sequencing, and bioinformatics technologies, it has become possible to identify genes differentially expressed between various physiological and pathological conditions that could serve as diagnostic biomarkers or treatment targets. Park et al. identified mRNAs and miRNAs differentially expressed among AMI stages in mice and suggested that ferroptosis during AMI inhibited the production of the cytoprotective antioxidant glutathione peroxidase 4 (GPX4), leading to oxidative stress, cardiomyocyte death, and loss of cardiac function [7]. In addition, through microarray analysis of infarcted mouse left ventricular myocardia, Chen et al. identified *Ccr1*, *Cxcl2*, *Ptgs 2*, and *Mmp8* as potentially critical genes for cardiac remodeling following AMI [8].

In addition to genes differentially expressed within infarcted regions, several studies have reported transcriptional differences between the infarcted myocardium and the adjacent non-infarcted myocardium [9], which suggests that the two regions may have different remodeling processes. For instance, Stuart et al. found that interstitial fibrosis in non-infarcted tissue is more arrhythmogenic than the compact scar in infarcted tissue [10], suggesting that myocardial cells in non-infarcted areas are more likely to induce post-AMI complications and thus increase the risk of fatal progression [9]. Therefore, it is essential to identify biomarkers or therapeutic targets in pathogenic non-infarcted myocardia after AMI. However, most studies have focused on the infarcted region or the entire left ventricular myocardium.

In the current study, we screened two microarray datasets (GSE775 and GSE19322) from the GEO database and one RNA-seq dataset (GSE110209) for DEGs between non-infarcted myocardia in C57BL/6J, MRL/MpJ, and FVB/N mice following AMI or sham treatment. We then identified DEGs uniformly altered across strains as potential diagnostic biomarkers or AMI treatment targets. A protein–protein interaction (PPI) network was constructed to identify hub genes most strongly associated with cardiac remodeling in non-infarcted myocardia post-AMI. Hub genes were then subjected to gene ontology (GO) and Kyoto Encyclopedia of Genes and Genomes (KEGG) analyses to reveal enriched biological functions and pathways. Finally, scRNA data analysis was conducted to examine the contribution of each hub gene to myocardial remodeling.

## 2. Materials and Methods

### 2.1. Gene Expression Profile Data

The GEO (https://www.ncbi.nlm.nih.gov/gds/ (accessed on 2 June 2022)) [11] is a public database for storing and accessing microarray datasets and high-throughput genomics data. To identify DEGs in the non-infarcted region after AMI, we screened 2 microarray datasets, GSE775 and GSE19322 and 1 RNA-seq dataset, GSE110209, which contained data from C57BL/6J, MRL/MpJ, and FVB/N mouse strains, respectively. In total, the GEO included 27 individual samples from the non-infarcted left ventricle, 14 acquired within 1 week after AMI and 13 acquired within 1 week after sham operation. We reorganized all samples’ data into four groups (GSE775-C57, GSE19322-C57, GSE19322-MRL, and GSE110209-FVB) according to strain origin. The GSE775-C57 group contained 3 AMI samples and 3 sham-operation samples from C57BL/6J mice, the GSE19322-C57 group contained 4 AMI samples and 3 sham-operation samples from C57BL/6J mice, the GSE19322-MRL group contained 4 AMI samples and 4 sham-operation samples from MRL/MpJ mice, and the GSE110209-FVB group contained 3 AMI samples and 3 sham-operation samples from FVB/N mice. Each group contained data from a single dataset, thereby avoiding batch effects. Detailed information about these groups is presented in Table 1. In a further step, the expression levels of the screened hub genes were verified in various types of cells of the infarcted remote myocardia using GSE183168 (RNA-seq dataset) and E-MTAB-7376 (single-cell sequencing dataset, obtained from total interstitial cell population) and the reliability of the hub genes was validated in the end using GSE119857 (RNA-seq dataset). All screened samples’ data were obtained from remote non-infarcted myocardia and did not contain the infarcted border.

### 2.2. Screening of Differentially Expressed Genes (DEGs)

The DEGs between AMI and sham-operation samples were analyzed using the “Limma” package of R 4.0.3 [12], with |log_2_FC| > 1.0 and *p* < 0.05 used as the criteria for statistical significance. Subsequently, these DEGs that were consistently and significantly up-regulated or down-regulated in all 4 groups (i.e., across strains) were identified using the “RobustRankAggreg” package, with |log_2_FC| > 1.0 and *p* < 0.05 (corrected for false discovery rate) as the criteria for significance.

### 2.3. PPI Network Construction and Screening of Modules

A protein-protein interaction (PPI) network was constructed using String version 11.0b (https://string-db.org/ (accessed on 2 June 2022)) and Cytoscape (version 3.7.1). Based on this PPI network, the Cytoscape plug-in MCODE was used to identify PPI modules with a score > 4.5. The DEGs were ranked according to their degree values and the top 10 DEGs were defined as hub genes.

### 2.4. GO and KEGG Pathway Enrichment Analyses

GO and KEGG pathway enrichment analyses were performed using the “clusterProfiler” package [13] of R with *p* < 0.05 as the cut-off criterion and results were visualized by the “pathview” package [14] of R.

### 2.5. Processing and Clustering scRNA-seq Data

Bioinformatics processing and clustering of the scRNA-seq data was performed in R using the “Seurat” package [15] with tSNE plots generated using “ggplot2”. The marker genes of clustered cell populations are referenced to the CellMarker database (http://bio-bigdata.hrbmu.edu.cn/CellMarker/ (accessed on 2 June 2022)).

## 3. Results

### 3.1. Identification of DEGs in Non-Infarcted Myocardia Following AMI

**Figure 1:**
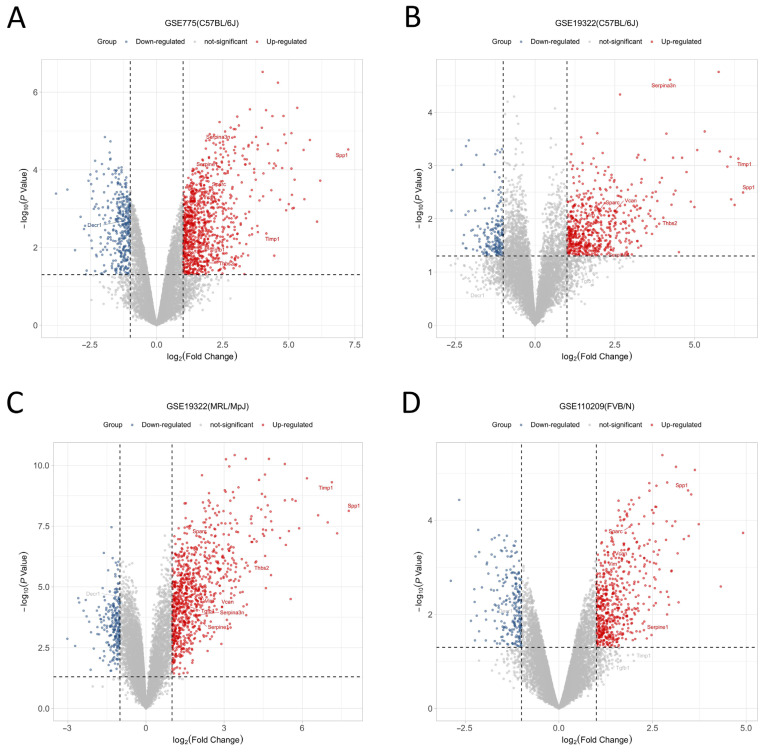
Volcano plot of DEGs from the 4 groups. (**A**) Volcano plot of group GSE775-C57; (**B**) Volcano plot of group GSE19322-C57; (**C**) Volcano plot of group GSE19322-MRL; (**D**) Volcano plot of group GSE110209-FVB; Blue points represent down-regulated genes, red points represent up-regulated genes, and gray points represent genes showing no significant difference in expression between conditions (threshold for significance: |log_2_FC| > 1.0 and *p* < 0.05). Hub genes are highlighted.

To identify DEGs in non-infarcted mouse myocardia following AMI, three gene expression profiles in GEO comparing non-infarcted myocardia between AMI and sham control groups, GSE775, GSE19322, and GSE110209, were first reorganized into four new data groups according to strain origin to eliminate strain-related DEGs (Table 1). Then, groups were screened for AMI-associated DEGs using the “Limma” package of R 4.0.3 with a significance threshold of |log_2_FC| > 1.0 and *p* < 0.05. Screening yielded 4183 DEGs in total, including 3110 up-regulated and 1073 down-regulated genes (Table 2 and Figure 1).

### 3.2. Identification of Genes Uniformly Up- or Down-Regulated across Strains

**Figure 2:**
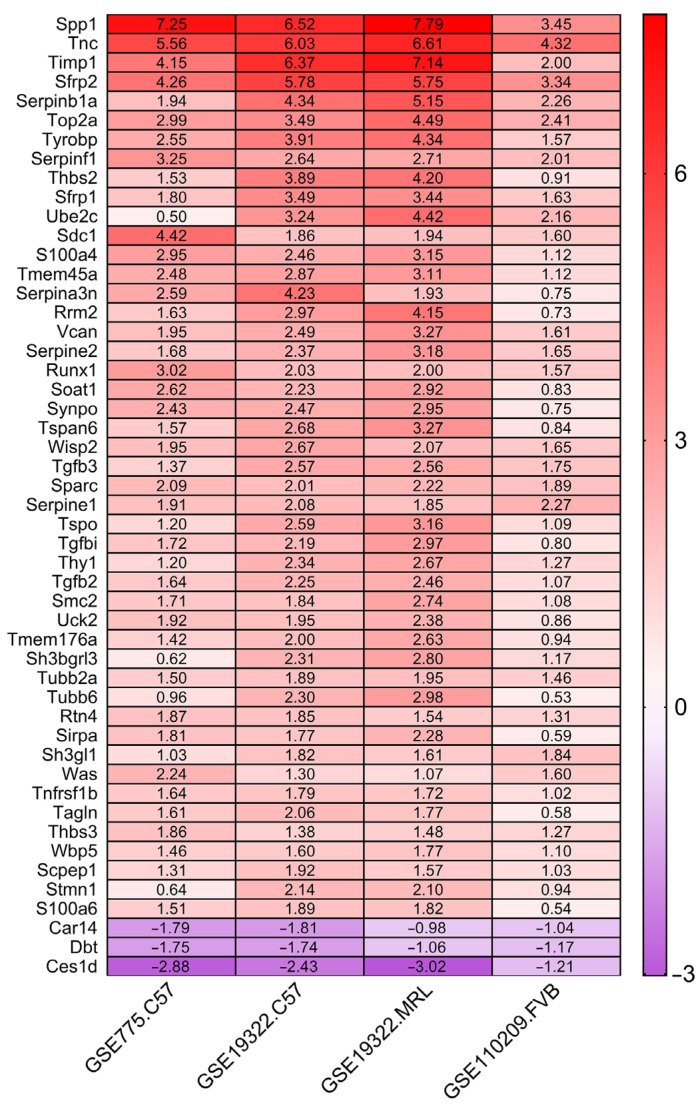
Heatmap of the top 50 DEGs. Each column represents one group and each row indicates one DEG showing uniform up-regulation (red) or down-regulation (purple) across mouse strains. The color shade indicates the magnitude of differential expression (logFC value).

All DEGs in the 4 groups were further screened for uniform up- or down-regulation using the “RobustRankAggreg” package of R and the criteria |log_2_FC| > 1.0 and *p* < 0.05 (fdr-corrected), which identified 110 genes that were uniformly up-regulated and 25 that were down-regulated in all strains (Table 3). The top 50 DEGs according to |log_2_FC| are presented in descending order as a heat map in Figure 2.

### 3.3. Construction of a PPI Network, Module Analysis, and Screening of Key Genes

**Figure 3:**
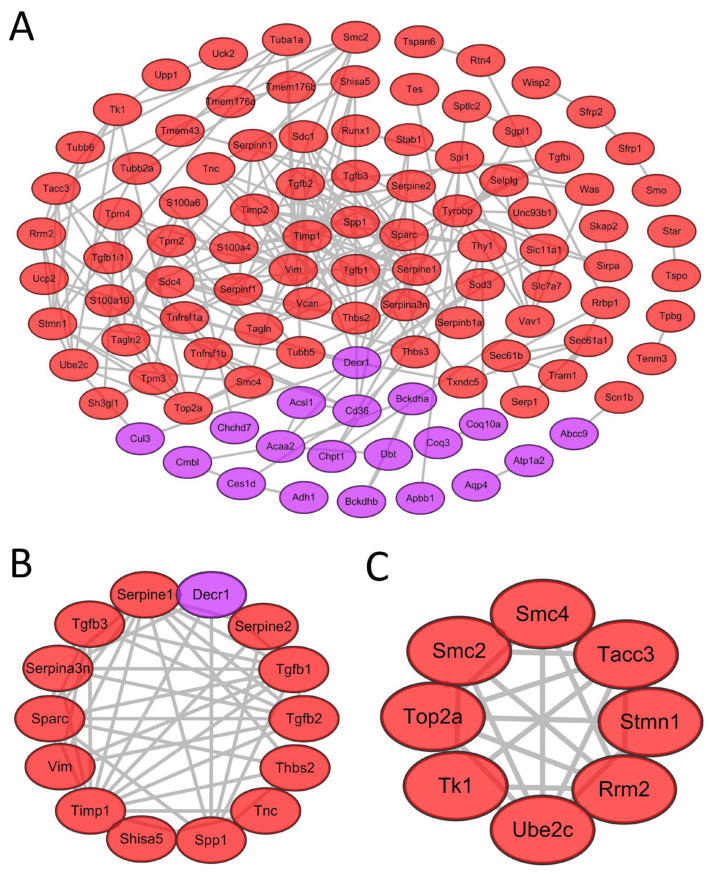
PPI network analysis and module analysis. (**A**) PPI network of uniformly regulated DEGs contains 105 nodes, 239 edges, and 2 modules. (**B**) Network module 1 contains 14 gene nodes and 51 edges (MCODE score = 7.846). (**C**) Network module 2 contains 8 gene nodes and 24 edges (MCODE score = 6.857). Red color represents up-regulated genes and purple represents down-regulated genes. MCODE, molecular complex detection (plug-in of Cytoscape).

The STRING database was used to construct a PPI network based on the 135 uniformly regulated genes. The final network included 105 nodes and 239 edges encompassing 19 down-regulated and 86 up-regulated genes (Figure 3A). Utilizing the MCODE plug-in, two significant modules (score > 4.5) were extracted from the PPI network. Module 1 contained 14 gene nodes (including 13 up-regulated genes and 1 down-regulated gene) and 51 edges (Figure 3B), while Module 2 contained 8 gene nodes (all up-regulated genes) and 24 edges (Figure 3C). Sorted by connectivity (degree) from high to low, we screened the top 10 hub genes, including *Timp1*, *Sparc*, *Spp1*, *Tgfb1*, *Decr1*, *Vim*, *Serpine1*, *Serpina3n*, *Thbs2*, and *Vcan* (Table 4). Degree, expression changes, and related literature for the top 50 genes are listed in Supplementary Materials S2.

### 3.4. GO and KEGG Enrichment Analyses

**Figure 4:**
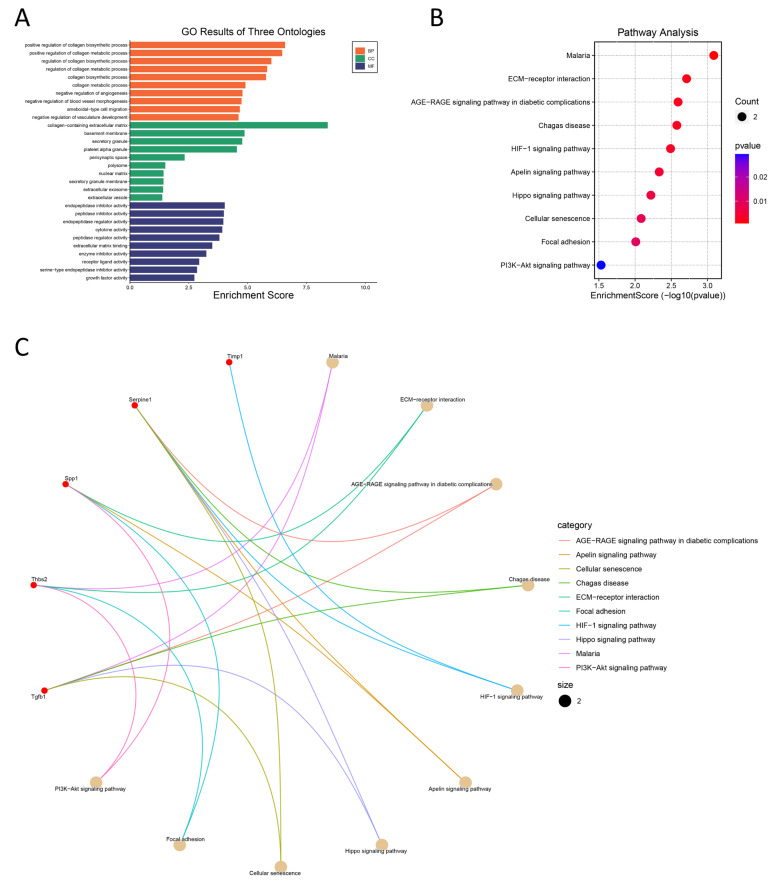
GO and KEGG enrichment analyses. (**A**) Histogram of the top 10 GO enrichment terms for each GO category. (**B**) Bubble of enriched KEGG pathways. (**C**) Relationship between hub genes and KEGG pathway.

To examine the biological functions of hub genes, GO and KEGG enrichment analyses were performed using the “clusterProfiler” package of R. The GO database annotates each gene according to three aspects: biological process (BP), cellular component (CC), and molecular function (MF) [16]. The top 10 GO terms for each aspect are shown in Figure 4. According to BP enrichment analysis, hub genes are involved in the “regulation of collagen biosynthetic and metabolic process”, “regulation of angiogenesis and development”, and “ameboidal-type cell migration”. Cell component enrichment analysis revealed that hub genes are associated with “extracellular matrix”, “basement membrane”, “polysome”, “nuclear matrix”, and “exosome”. Finally, according to MF analysis, hub genes are involved in “peptidase regulator activity”, “receptor ligand activity”, “cytokine activity”, and “extracellular matrix structural constituent”. The results of KEGG pathway analysis for hub genes are summarized and plotted in Figure 4B and Figure 4C and they are associated with “ECM-receptor interaction”, “AGE-RAGE signaling pathway”, “HIF-1 signaling pathway”, “adipocytokine signaling pathway”, “Apelin signaling pathway”, “Hippo signaling pathway”, and multiple disease pathways.

### 3.5. Expression of Hub Genes in Different Cell Populations

**Figure 5:**
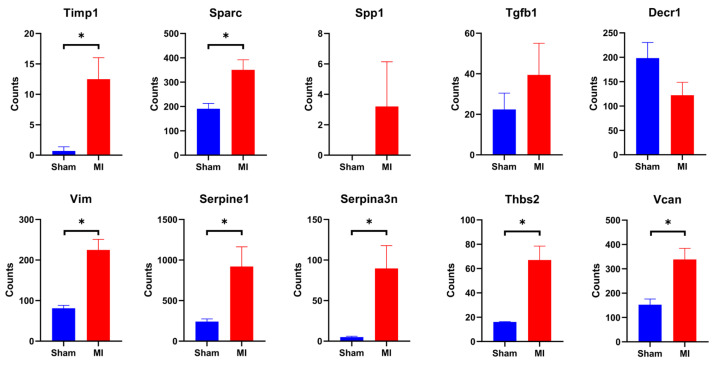
Hub gene expression in cardiomyocytes. MI indicates infarction surgery group, Sham indicates sham surgery group. Error lines indicate SEM. * *p* < 0.05, Mann-Whitney U test.

**Figure 6:**
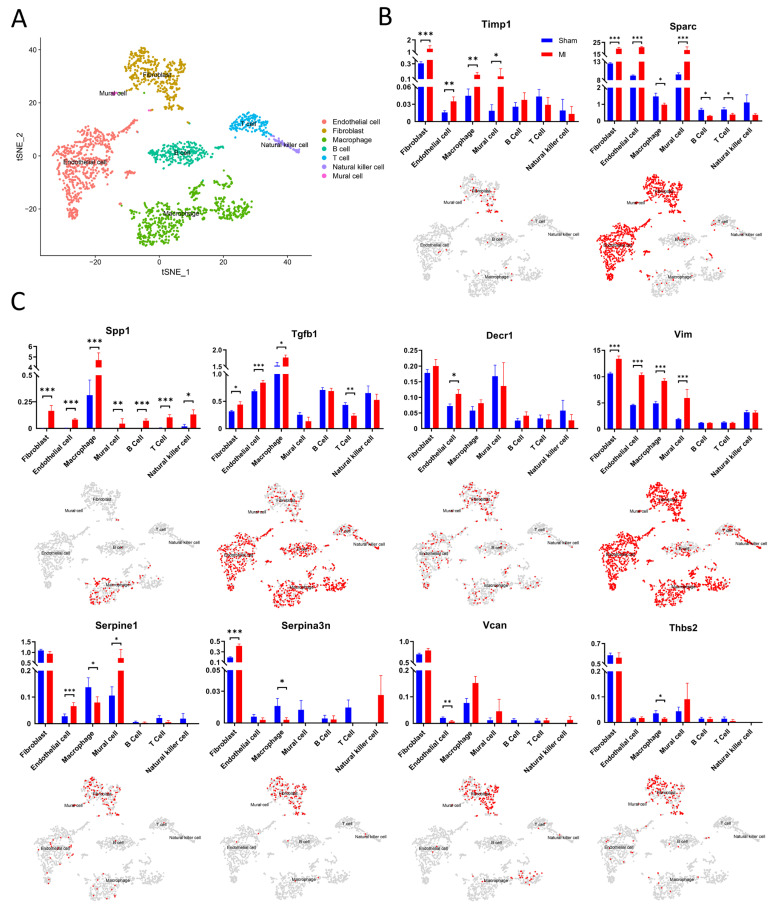
Single-cell sequencing of TIP from hearts after myocardial infarctions. (**A**) t-SNE plots of aggregated cardiac TIP 7d after infarction surgery; (**B**,**C**) expression of hub genes in different populations and localization in t-SNE plots. Blue color indicates sham surgery group, red indicates infarction surgery group. * *p* < 0.05, ** *p* < 0.01, *** *p* < 0.001, Mann-Whitney U test.

To further explore the pathways of the top 10 hub genes involved in regulating the repair process after myocardial infarction, we analyzed the expression of hub genes in various cell populations after myocardial infarction by RNA-seq (GSE183168) and single-cell sequencing (E-MTAB-7376) data. From the RNA-seq data, we extracted the expression data of hub genes in cardiomyocytes from non-infarcted myocardia to compare to the sham-operation group (Figure 5). In addition, in single-cell sequencing, the total cardiac interstitial cell population (TIP) from the hearts of C57 mice at 7d post-sham or MI surgery was isolated and sequenced. We performed clustering on an aggregate of cells using the “Seurat” R package [15], with cell populations visualized in t-SNE dimensionality reduction plots. A total of seven classes of cell populations were identified (Figure 6A), including fibroblast (marked by *Ckap4, Col1a2, Col3a1, and Mmp2*), macrophage (*Itgam, Lgals3, and Lyz1*), endothelial cell (*Egfl7, Emcn, and Flt1*), mural cell (*Gjc1, Higd1b, and Ifitm1*), B cell (*Cd79a, Cd79b, Ly6d, and Mzb1*), T cell (*Cd8a, Cd8b1, and Cd3g*), and natural killer cell (*Klra8, Klrb1c, Klrc1, and Ncr1*). Furthermore, the expression of hub genes was extracted and used to perform differential analysis for different cell populations (Figure 6B,C). Combining the results of transcriptome data analysis with those of the single-cell sequencing data, we found that the expression levels of hub genes were significantly different in cell populations of the MI group compared to the sham-operation group, which was also consistent with the results of differential analysis in the previous paper, demonstrating that hub genes play key roles in the remodeling process after myocardial infarction.

It has been shown that cardiac fibroblasts, which account for approximately 10% of all cardiac cells, are involved in inflammation and phagocytosis and play a major role in cardiac repair [17,18,19]. Macrophages dominate the inflammatory process after injury and the timely resolution of inflammation is necessary to limit fibrosis and achieve tissue replacement, while uncontrolled inflammation can lead to negative progressions, such as increased fibrosis and ventricular wall sclerosis [20,21]. Our results show that, 7d after myocardial infarction, the expression levels of *Timp1, Sparc, Spp1, Tgfb1, Vim,* and *Serpina3n* were significantly increased in fibroblasts, and in macrophages, the expression levels of *Timp1, Spp1, Tgfb1,* and *Vim* were significantly increased; in contrast, *Sparc, Serpine1, Serpina3n,* and *Thbs2* were significantly down-regulated. This result demonstrates that *Timp1, Sparc, Spp1, Tgfb1, Vim, Serpina3n, Serpine1,* and *Thbs2* are involved in the regulation of cardiac remodeling by fibroblasts and macrophages. Notably, *Sparc* was significantly differentially expressed in all cell types except natural killer cells after infarction, while *Spp1* was significantly differentially expressed in all cell types except cardiomyocytes, which also indicates that *Sparc* and *Spp1* are involved in many cellular regulatory processes during cardiac remodeling as a crucial role.

## 4. Discussion

Despite major advances in treatment (such as percutaneous coronary intervention, fibrinolysis, antiplatelet drugs, and anticoagulants), AMI is still the main cause of premature mortality worldwide [3,6]. Recent microarray analysis of patient peripheral blood samples and infarcted myocardium samples from model mice have also identified diagnostic biomarkers and novel treatment targets [8,22,23]. In contrast, there are few reliable diagnostic markers or therapeutic targets for pathogenic but non-infarcted myocardia following AMI.

**Figure 7:**
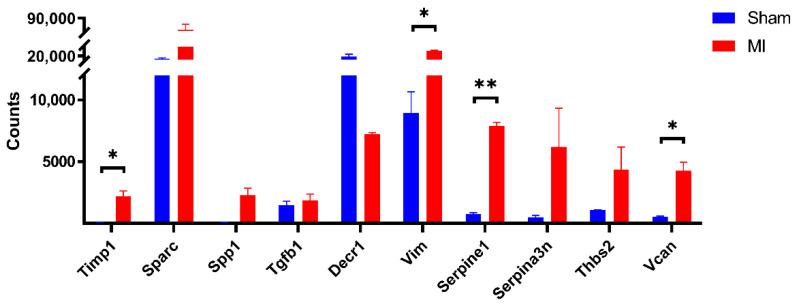
Hub gene expression in cardiomyocytes in GSE119857. MI indicates infarction surgery group, Sham indicates sham surgery group. Error lines indicate SEM. * *p* < 0.05, ** *p* < 0.01, Mann-Whitney U test.

In this study, 135 DEGs uniformly up-regulated or down-regulated across mouse strains following experimental AMI (compared to sham controls) were identified through comprehensive bioinformatics analysis and 10 strongly interlinked hub genes (*Timp1, Sparc, Spp1, Tgfb1, Decr1, Vim, Serpine1, Serpina3n, Thbs2*, and *Vcan*) were identified by the construction of a PPI network from these DEGs. Among them, the expression of *Timp1, Sparc, Spp1, Tgfb1, Vim, Vcan, Serpina3n, Serpine1,* and *Thbs2* was up-regulated and *Decr1* expression was down-regulated in remote non-infarcted myocardia after AMI. Finally, the differential expression of the 10 hub genes in additional GEO datasets (GSE119857), which performed high-throughput RNA-seq of the remote myocardium 7 days after infarction, was consistent with our initial results (Figure 5 vs. Figure 7), substantiating the importance of the identified 10 hub genes.

According to the previous analysis, hub genes were involved in the extracellular matrix (ECM)-receptor interaction pathway and were closely associated with fibroblast and macrophage regulation of cardiac remodeling processes. It is shown that activated fibroblasts are the main source of structural ECM proteins in fibrotic hearts [24]. In addition, we also noted that *Decr1* is a mitochondrial enzyme involved in polyunsaturated fatty acid metabolism [25], which provides another possibility for hub genes to regulate cardiac remodeling. Collectively, these findings suggest that the hub genes influence the ventricular remodeling of non-infarcted tissue by modulating the ECM-mediated myocardial fibrosis, macrophage-driven inflammation, and fatty acid metabolism. Among the top 50 DEGs, most of the genes have been studied for association with AMI except for the 10 hub genes but there are still 14 genes for which no study has confirmed their association with AMI (already labeled as “None” in Supplementary Materials S2). Of interest, it was recently reported that Shisa5 can block spontaneous contact between endoplasmic reticulum exit sites and phagophore and thus inhibit autophagy [26]. The balance between autophagy and apoptosis is important in cell survival and cardiac function after AMI [27]. Therefore, whether the up-regulation of Shisa5 expression after AMI is involved in the LV remodeling process by inhibiting autophagy is worthy of deeper investigation, which would also provide new insights into the biomarkers of the autophagic process after AMI.

### 4.1. Extracellular Matrix (ECM)-Mediated Myocardial Fibrosis

The cardiac ECM facilitates molecular and electrical signaling among cardiomyocytes and provides mechanical support to the myocardium [28,29]. The main components of the ECM are structural proteins, non-structural intercellular signaling proteins (ligands and receptors), proteases, and protease inhibitors [28]. After AMI, ECM structure and composition are altered in both infarcted and non-infarcted zones [30], which, in turn, can alter the gross shape and function of the left ventricle (LV) [30,31]. Optimal LV remodeling (i.e., for repair) is the proper balance between ECM synthesis and degradation [28]. Excessive ECM accumulation causes diastolic dysfunction by increasing myocardial stiffness and reducing compliance, whereas inadequate ECM deposition promotes progressive infarct wall weakening and enlargement, which can lead to LV dilation, aneurysm development, and rupture [28,32]. An imbalance between ECM synthesis and degradation may thus be the primary pathomechanism for cardiac rupture, a dramatic and fatal complication following AMI [33]. Our results show that the expression of hub genes were mainly significantly up-regulated instead of down-regulated, which also presented in fibroblasts, reflecting that ECM-mediated myocardial fibrosis process may play a major role in LV repair after AMI.

Many of the identified hub genes were expressed in cardiac fibroblasts and implicated in the formation or regulation of the ECM, including Versican (*Vcan*) [32], Thrombospondin 2 (*Thbs2*), Secreted acidic cysteine-rich glycoprotein (*Sparc*), Osteopontin (*Spp1*), Serine (or cysteine) peptidase inhibitor I (*Serpine1*) [33], Serine (or cysteine) peptidase inhibitor 3N (*Serpine3n*) [34], Transforming growth factor-beta 1 (*Tgfb1*), and Tissue inhibitor of metalloproteinase 1 (*Timp1*) [35]. Neutrophils and macrophages enter the infarct site in response to the release of damage signals and cytokines. In turn, infiltrating macrophages and neutrophils release various inflammatory mediators and matrix metalloproteinases (MMPs) that break down the ECM to facilitate the phagocytosis of necrotic tissue [28,36]. To maintain homeostatic control of ECM degradation, injured tissues also up-regulate the expression of the tissue inhibitors of metalloproteinases (TIMPs) [28,32]. In fact, TIMP-1 loss impaired LV contractile performance in mice following AMI and hastened the development of LV pump failure. Moreover, TIMP-1 expression level was correlated with echocardiographic indicators of LV size and dysfunction [37,38].

*Spp1* [39] and *Thbs2* [33,40] are also implicated in remodeling by regulating the expression and activity of MMPs. *Spp1* up-regulates tissue inhibitors of MMPs and collagen, and conversely down-regulates MMP-1 expression in cardiac fibroblasts [39,41]. It has also been suggested that *Spp1*, acting via β3 integrins, inhibits interleukin-1beta (IL-1β)-induced activation of MMP-2 and MMP-9, thereby increasing the deposition of collagen after AMI [42]. Furthermore, MMP-2 activation has been linked to a reduction in heart tissue tensile strength as well as impaired systolic and diastolic dysfunction [43]. The function of *Thbs2* in cardiac fibrosis induced by pressure overload has been widely studied. Following angiotensin II therapy, *Thbs2* null mice demonstrated substantial interstitial edema and cardiomyocyte injury, both of which were linked to elevated MMP-2 and MMP-9 activity. Thus, the primary function of *Thbs2* in myocardial remodeling appears to be the conservation of ECM integrity through the inhibition of MMP activity [40]. In addition, *Thbs2*-KO mice exhibited increased expression of miR-29 in the skin, accompanied by reduced fibrous collagen and LOX-mediated fibrous collagen cross-linking, resulting in a markedly altered ECM structure [44].

TGF-β is a critical modulator of inflammation, fibrotic healing, and other cellular reparative processes post-infarction [45,46]. In the early phase post-AMI, TGF-β may suppress inflammation by deactivating macrophages and decreasing chemokine and cytokine expression [45]. However, at a later stage TGF-β may contribute to left ventricular remodeling by inducing ECM changes and activating fibrogenic pathways in non-infarcted myocardia [45]. Several studies have suggested that *Tgfb1* increases ECM protein production and heart hypertrophy in response to Ang II [47,48,49,50] through a mechanism involving *Spp1* [51,52], *Serpine1* [53,54], and *Sparc* [55,56]. Serpine1 inhibits urokinase-type plasminogen activator (uPA), which controls the generation of plasmin (Pm) [53]. In addition, Pm has been linked to the activation of TGF-β [53,57]. Although *Serpine1* is a known downstream transcription target of TGF-β, in the Ang II infusion model of post-AMI remodeling, *Serpine1* deficiency resulted in a dramatic up-regulation of TGF-β, integrin-β, and ECM-associated protein transcripts [54]. These findings strongly suggest that Serpine1 has a seminal role in maintaining cardiac ECM integrity and cardiomyocyte integrin–matrix interactions, as well as in modulating cardiac TGF-β. Similarly, Schellings and colleagues found that SPARC increased TGF signaling in cardiac fibroblasts and altered the ECM architecture, processes that are essential for appropriate infarct healing and collagen maturation as well as for preventing heart rupture, dilatation, and dysfunction following AMI [55].

### 4.2. Macrophage-Driven Inflammation

Following AMI, signals from damaged myocardia attract circulating monocytes, which then develop into macrophages [58,59]. The initial inflammatory response to damage, subsequent wound healing, and tissue homeostasis are all dependent on macrophages [60] and several studies have reported that the absolute number of macrophages is elevated in the remote non-infarcted myocardium even at 8 weeks after AMI [60,61]. Furthermore, maladaptive macrophage activity can lead to ventricular remodeling. In the early days following AMI, inflammatory M1 macrophages secrete cytokines, chemokines, and TGF-β that initiate tissue regeneration, while anti-inflammatory M2 macrophages regulate this process [58,62]. However, chronic M1 macrophage activity can prolong the pro-inflammatory phase, thereby promoting an enlargement of the infarcted area, delaying tissue repair and scar tissue development driven by M2 macrophages, and aggravating unfavorable LV remodeling [58]. Therefore, regulating the M1-M2 macrophage balance may be an effective strategy for limiting infarct size and preventing undesirable LV remodeling.

Many factors regulate the proliferation, recruitment, polarization, and anti-inflammatory activities of macrophages. As a specific component of the provisional ECM, *Vcan* may be involved in activating macrophages and inducing inflammatory cytokine release [63]. Reactive oxygen species (ROS) promote pro-inflammatory signaling by macrophages and several studies have suggested that *Vim* suppresses ROS production [64,65]. Alternatively, *vimentin* deficiency caused poor endocytosis, increased oxidative stress, and elevated CD36 expression in macrophages [66]. In addition, *Sparc*-null mice exhibited decreased macrophage numbers in the remote region after AMI, suggesting that SPARC may modulate macrophage viability and chronic immunological responses [55,67]. The significant down-regulation of Sparc expression in macrophages (Figure 6B) demonstrated its inhibitory effect on the anti-inflammatory activity of macrophages, which, together with *Spp1*, whose expression was significantly up-regulated (Figure 6C), regulated the homeostasis of anti-inflammatory activity. Interestingly, recent studies have found that the activation of CD4T cells in the early stages of AMI promoted the inflammatory response and later declined to ensure immune regression, similar to the role of macrophages [68,69,70]. This trend was also consistent with the expression of *Sparc* and *Spp1* in macrophages and T cells, suggesting that *Sparc* and *Spp1* may also regulate the balance of the pro-inflammatory activity of T cells after AMI.

### 4.3. Fatty Acid Metabolic Pathways

**Figure 8:**
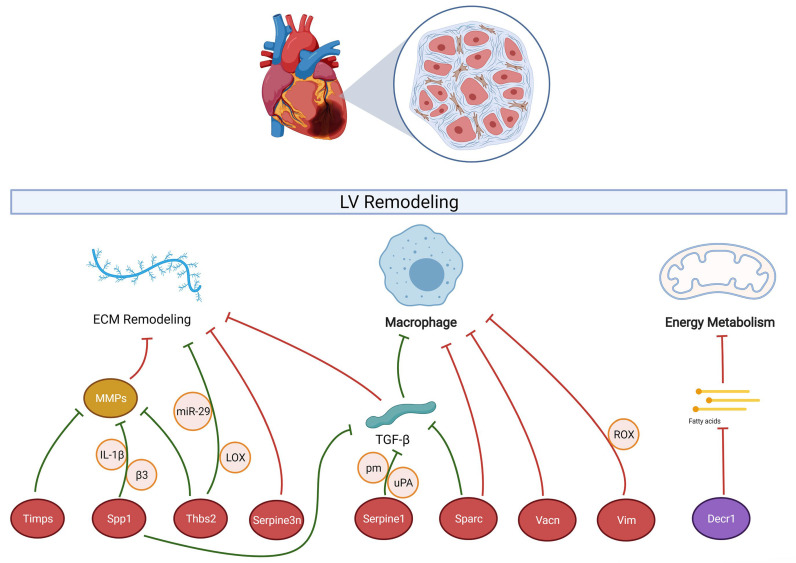
Mechanisms of myocardial remodeling after AMI by the 10 hub genes. Up-regulated hub genes are indicated by red ovals and down-regulated hub genes are indicated by purple ovals. Red arrows represent positive regulation and green arrows represent negative regulation. Circles represent intermediate or interacting factors in the regulatory process. “MMPs”, “TGF-β”, and “Fatty acids” are regulatory mediators, while “ECM remodeling”, “Macrophage”, and “Energy Metabolism” are regulatory pathways.

Heart failure is a common complication after AMI. This condition is characterized by abnormalities in energy metabolism, such as mitochondrial dysfunction and decreased fatty acid (FA) oxidation, which can be partially compensated by increased glucose metabolism [71]. This metabolic change may represent an adaptive mechanism as it can reduce oxygen consumption and concomitant ROS generation. However, glucose oxidation produces less ATP than FA oxidation, which may increase contractile dysfunction and impair reparative LV remodeling after AMI [72]. Therefore, metabolic interventions that support heart energy conversion may be important adjuvant treatments for heart failure [71]. *Decr1* is a mitochondrial enzyme implicated in the metabolism of polyunsaturated fatty acids [25,73]. Our results (Table 3 and Figure 5) indicate that *Decr1* expression was down-regulated both at the tissue level and in cardiomyocytes after AMI, while multiple studies have shown that *Decr1* expression was down-regulated in cardiovascular disease [25,74,75], which is consistent with the observed reduction in FA oxidation after AMI, suggesting that *Decr1* may be a potential target for metabolic intervention. A schematic representation of the regulatory network formed by these 10 hub genes after AMI is shown in Figure 8.

## 5. Conclusions

In summary, we identified 10 hub genes (*Timp1, Sparc, Spp1, Tgfb1, Decr1, Vim, Serpine1, Serpina3n, Thbs2* and *Vcan)*, that may directly or indirectly regulate myocardial remodeling in the non-infarcted zone after AMI by altering ECM composition, macrophage activity, and fatty acid metabolism. This study thus provides many novel potential biomarkers for the improvement and treatment targets for AMI and associated complications.

## Figures and Tables

**Table 1:** Compositions of individual datasets.

Group Name	Datasets	Strain	Sample	AMI	Sham
GSE775-C57	GSE775	C57BL/6J	niLV	3	3
GSE19322-C57	GSE19322	C57BL/6J	niLV	4	3
GSE19322-MRL	GSE19322	MRL/MpJ	niLV	4	4
GSE110209-FVB	GSE110209	FVB/N	niLV	3	3

Abbreviations: AMI, counts of AMI samples; niLV, no infected left ventricular; Sham, counts of sham-operation samples.

**Table 2:** Differentially expressed genes in non-infarcted mouse myocardia post-AMI compared to sham controls.

Data Group Name	Total DEGs	Up-Regulated	Down-Regulated
GSE775-C57	1286	933	353
GSE19322-C57	782	593	189
GSE19322-MRL	1113	886	227
GSE110209-FVB	1002	698	304

**Table 3:** Genes uniformly up-regulated or down-regulated across strains following AMI.

Expression Level	Gene Name
Up-regulated	*Spp1, Tnc, Timp1, Sfrp2, Serpinb1a, Top2a, Tyrobp, Serpinf1, Thbs2, Sfrp1, Ube2c, Sdc1, S100a4, Tmem45a, Serpina3n, Rrm2, Vcan, Serpine2, Runx1, Soat1, Synpo, Tspan6, Wisp2, Tgfb3, Sparc, Serpine1, Tspo, Tgfbi, Thy1, Tgfb2, Smc2, Uck2, Tmem176a, Sh3bgrl3, Tubb2a, Tubb6, Rtn4, Sirpa, Sh3gl1, Was, Tnfrsf1b, Tagln, Thbs3, Wbp5, Scpep1, Stmn1, S100a6, Sec61a1, Stab1, Slc39a6, Tgfb1, Tacc3, Tubb5, S100a10, Tagln2, Slc11a1, Tmem43, Slc20a1, Tenm3, Tmed3, Vim, Star, Skap2, Timp2, Tmem176b, Ucp2, Sgpl1, Ugdh, Txndc5, Trpv2, Tnfrsf1a, Spi1, Slc7a7, Sod3, Trim47, Selplg, Shisa5, Sat1, Tgif1, Smo, Sec61b, Snx10, Sdc4, Serp1, Tpm3, Scd2, Tpm4, Serpinh1, Vav1, Scn1b, Tgfb1i1, Sptlc2, Smc4, Sntb2, Tes, Rrbp1, Tpbg, Unc93b1, Tpm2, Zmat3, Zfp36l2, S100pbp, Srsf9, Tram1, St3gal2, Usp18, Upp1, Tk1, Tuba1a, Sema3f*
Down-regulated	*Crhr2, Akap8, Coq3, Adi1, Cul3, Chpt1, Cmtm8, Chchd7, Bckdha, Cmbl, Adh1, Acsl1, Bckdhb, Cd36, Acaa2, Coq10a, Apbb1, Decr1, Atp1a2, Abcc9, Aqp4, Dbp, Car14, Dbt, Ces1d*

**Table 4:** Detail information of top 10 hub genes.

Gene	Protein Name	Expression Change	Degree	Module (1 or 2)
*Timp1*	Tissue inhibitor of metalloproteinase 1	Up	18	1
*Sparc*	Secreted acidic cysteine-rich glycoprotein	Up	16	1
*Spp1*	Osteopontin	Up	16	1
*Tgfb1*	Transforming growth factor, beta 1	Up	16	1
*Decr1*	2,4-dienoyl CoA reductase 1	Down	15	1
*Vim*	Vimentin	Up	14	1
*Serpine1*	Serine (or cysteine) peptidase inhibitor, clade E, member 1	Up	13	1
*Serpina3n*	Serine (or cysteine) peptidase inhibitor, clade A, member 3N	Up	11	1
*Thbs2*	Thrombospondin 2	Up	11	1
*Vcan*	Versican	Up	10	Neither

## Data Availability

GSE775: accessed on 2 June 2022, https://www.ncbi.nlm.nih.gov/geo/query/acc.cgi?acc=GSE775; GSE19322: accessed on 2 June 2022, https://www.ncbi.nlm.nih.gov/geo/query/acc.cgi?acc=GSE19322; GSE110209: accessed on 2 June 2022, https://www.ncbi.nlm.nih.gov/geo/query/acc.cgi?acc=GSE110209; GSE183168: accessed on 2 June 2022, https://www.ncbi.nlm.nih.gov/geo/query/acc.cgi?acc=GSE183168; E-MTAB-7376: accessed on 2 June 2022, https://www.ebi.ac.uk/biostudies/arrayexpress/studies/E-MTAB-7376; GSE119857: accessed on 9 November 2022, https://ncbi.nlm.nih.gov/geo/query/acc.cgi?acc=GSE119857.

## Supplementary Materials

The following supporting information can be downloaded at: https://www.mdpi.com/article/10.3390/jcdd9120409/s1, Supplementary Materials S1: Results of GO and KEGG analysis of hub genes; Supplementary Materials S2: Research progress of Top50 DEGs [76,77,78,79,80,81,82,83,84,85,86,87,88,89,90,91,92,93,94,95,96,97,98,99,100,101].

## Abbreviations

AMI	Acute myocardial infarction
GEO	gene expression omnibus
DEG	differentially expressed gene
PPI	protein–protein interaction
GO	gene ontology
KEGG	Kyoto Encyclopedia of Genes and Genomes
ECM	extracellular matrix
TIP	total cardiac interstitial cell population
LV	left ventricle
