# Supplementary material for: Identification of Hub Genes in the Remodeling of Non-Infarcted Myocardium Following Acute Myocardial Infarction
Source: J Cardiovasc Dev Dis. 2022 Nov 22;9(12):409. doi: 10.3390/jcdd9120409 (PMC9788553; doi:10.3390/jcdd9120409)
Supplement: Supplementary file 1 [file jcdd-09-00409-s001.zip › Supplementary Materials S2.pdf]

Research progress of Top50 DEGs.

Gene	Expression Change	Degree	Ref. (AMI associated)
Timp1	Up	18	Hub Gene
Sparc	Up	16	Hub Gene
Spp1	Up	16	Hub Gene
Tgfb1	Up	16	Hub Gene
Decr1	Down	15	Hub Gene
Vim	Up	14	Hub Gene
Serpine1	Up	13	Hub Gene
Serpina3n	Up	11	Hub Gene
Thbs2	Up	11	Hub Gene
Vcan	Up	10	Hub Gene
Stmn1	Up	9	None
Tgfb2	Up	9	[76]
Tyrobp	Up	9	[77]
Sdc4	Up	8	[78]
Tgfb3	Up	8	[76]
Top2a	Up	8	None
Ube2c	Up	8	None
Rrm2	Up	7	[79]
Cd36	Up	7	[80,81]
Serpine2	Up	7	[82]
Tagln	Up	7	[83]
Tacc3	Up	7	None
Spi1	Up	7	[84]
Timp2	Up	7	[85]
Thy1	Up	7	[86]
Sdc1	Up	6	[87]
Smc2	Up	6	None
Tagln2	Up	6	[88]
Slc11a1	Up	6	None
Tk1	Up	6	[89]
Vav1	Up	6	[90]
Tubb5	Up	6	None
Tpm4	Up	6	[91]
Acaa2	Down	5	[92]
Shisa5	Up	5	None
S100a4	Up	5	[93]
Tuba1a	Up	5	None
Serpinb1a	Up	5	[94]
Smc4	Up	5	None
S100a6	Up	5	[95]
Tpm3	Up	5	[96]
Tnc	Up	5	[97]
Sec61a1	Up	4	[98]
Serpinh1	Up	4	[99]
Sec61b	Up	4	None
Tpm2	Up	4	None

Tgfbi	Up	4	[100]
Thbs3	Up	4	[101]
Chpt1	Down	3	None
Rrbp1	Up	3	None
